# Supplementary material for: Surrogate Virus Neutralisation Test Based on Nanoluciferase-Tagged Antigens to Quantify Inhibitory Antibodies against SARS-CoV-2 and Characterise Omicron-Specific Reactivity in a Vaccination Cohort
Source: Vaccines (Basel). 2023 Dec 8;11(12):1832. doi: 10.3390/vaccines11121832 (PMC10748151; doi:10.3390/vaccines11121832)
Supplement: Supplementary file 1 [file vaccines-11-01832-s001.zip › vaccines-2746251-supplementary.pdf]

vaccines

MDPI

Supplemental Figures

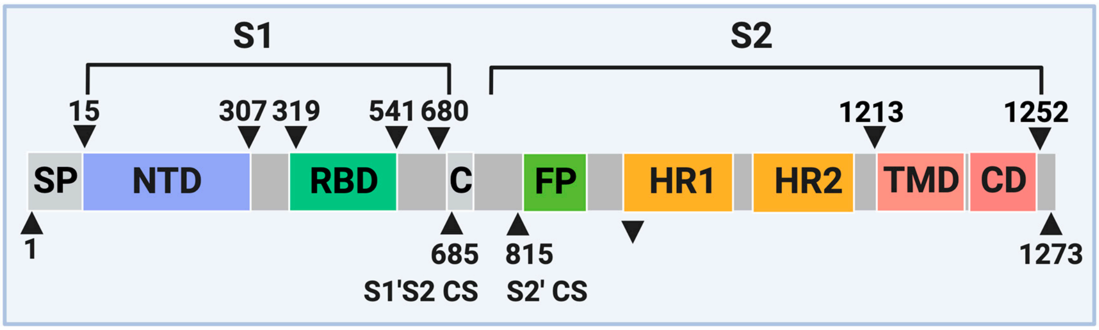

Supplemental Figure S1. SARS-CoV-2 spike protein primary structure. S1: spike protein domain 1, S2: spike protein domain 2, SP: signal peptide, NTD: N-terminal domain, RBD: receptor binding domain, C/CS: cleavage site, FP: fusion peptide, HR: heptad repeat, TMD: transmembrane domain, CD: cytodomain. The illustration was created with BioRender.com.

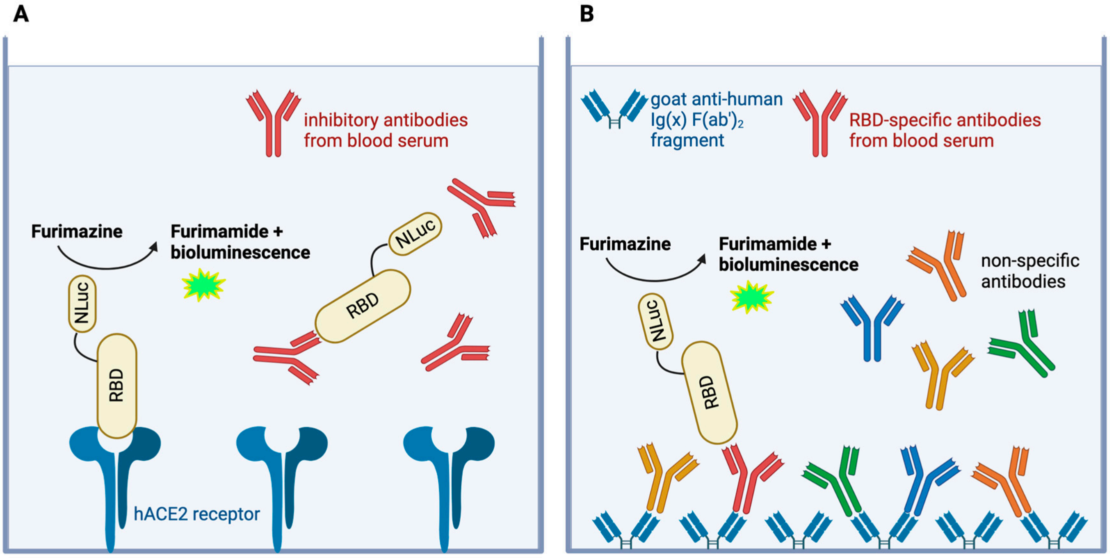

Supplemental Figure S2. Schematic illustration of the in-house surrogate virus neutralisation test (**A**) and the immunoglobulin capture enzyme immunoassay (**B**) designed to measure SARS-CoV-2-specific inhibitory antibodies in human serum samples using RBD-NLuc fusion proteins as diagnostic antigens. Serum antibody-mediated inhibition of RBD binding to hACE2 (**A**) and capture of RBD-NLuc fusion proteins by specific antibodies (**B**) were quantified using nanoluciferase-mediated bioluminescence signals. NLuc: nanoluciferase; RBD: receptor binding domain; hACE2: human angiotensin-converting enzyme 2. The illustration was created with BioRender.com.

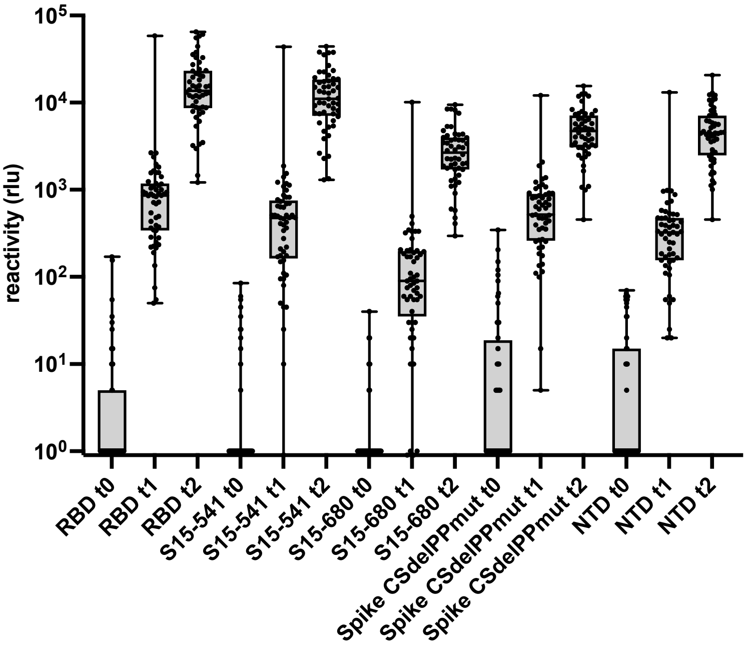

Supplemental Figure S3: Recognition of NLuc-tagged S protein constructs by human sera. Reactivity of constructs secNLuc-RBD (RBD), secNLuc-S15-541 (S15-541), secNLuc-15-680 (S15-680), secNLuc-spike-CSdel-PPmut (Spike CSdelPPmut) and secNLuc-NTD (NTD) was quantitated by gamma-chain capture EIA as described in Materials and Methods. 53 consecutive sera of vaccinees obtained immediately before vaccination (t0, open circles), four weeks after the first vaccination (t1, grey circles) and four weeks after the second vaccination (black circles) were tested at a dilution of 1:100. Reactivity is given as rlu. Values <0 were set to 1.

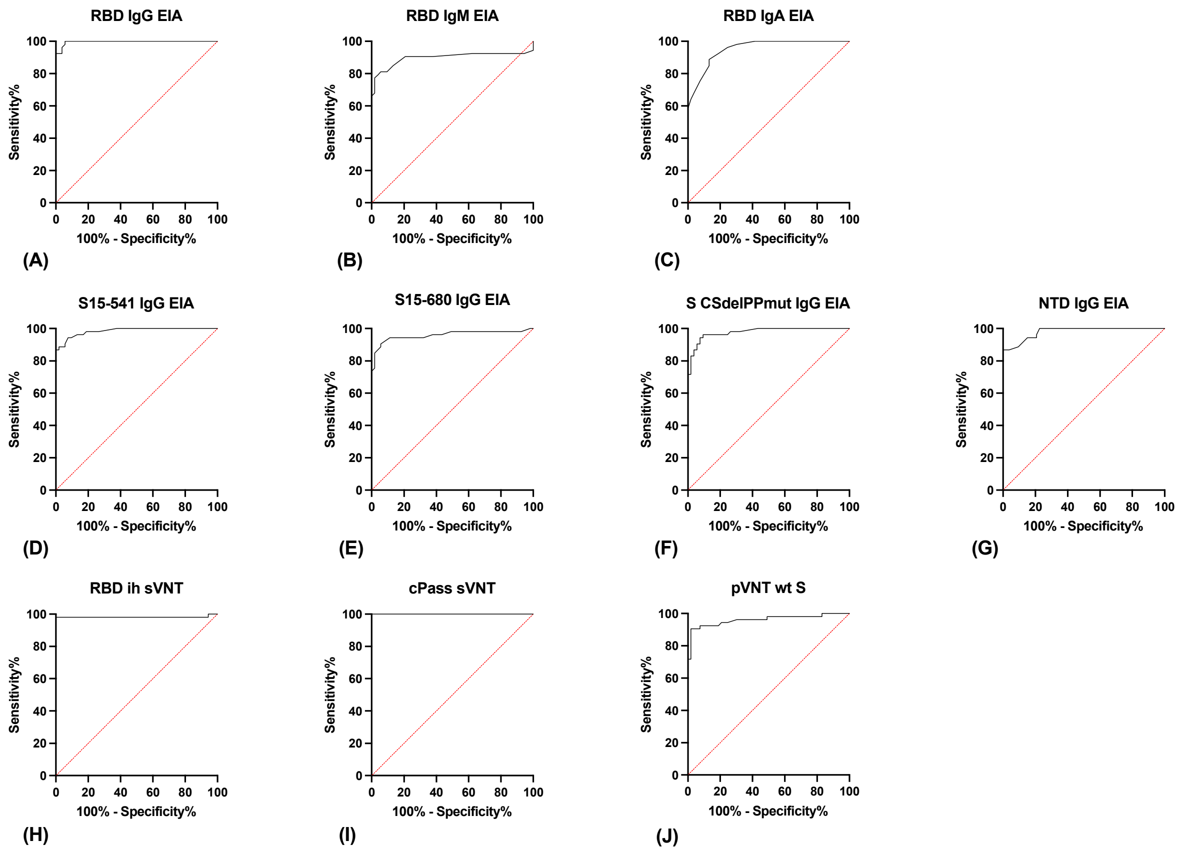

Supplemental Figure S4: Diagnostic performance of antigen constructs. Discrimination of serum samples obtained at t0 and t1 was determined by calculating ROC curves. (**A-C**) Reactivity of sera (n=53) with secNLuc-RBD in γ-capture EIA (**A**), μ-capture EIA (**B**) and α-capture EIA (**C**). (**D-G**) Reactivity in gamma-capture EIA with secNLuc-15-541 (**D**), secNLuc-15-680 (**E**), secNLuc-spike-CSdel-PPmut (**F**) and secNLuc-NTD (**G**). (**H-J**) reactivity of sera in in-house sVNT with secNLuc-RBD (**H**), commercial sVNT (cPass) (**I**), and pVNT with wt S (**J**).

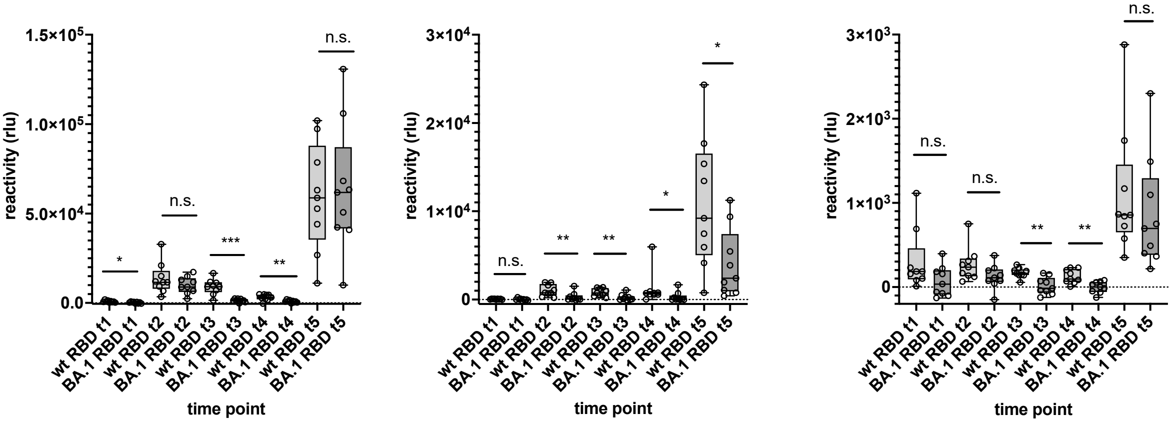

(A) (B) (C)

Supplemental Figure S5: Detection of antibodies reactive with the Omicron BA.1 variant in IgG EIA (**A**), IgA EIA (**B**) and IgM EIA (**C**). Horizontal lines represent the variable median. The significance of differences in reactivity at time points t1 to t5 was determined by the Mann-Whitney test (P >0.05: n.s., P <0.05: *, P <0.01: **, P <0.001: ***, P <0.0001: ****).
